# Cost-Effectiveness Analysis of Local Treatment in Oligometastatic Disease

**DOI:** 10.3389/fonc.2021.667993

**Published:** 2021-06-15

**Authors:** Dirk Mehrens, Marcus Unterrainer, Stefanie Corradini, Maximilian Niyazi, Farkhad Manapov, C. Benedikt Westphalen, Matthias F. Froelich, Moritz Wildgruber, Max Seidensticker, Jens Ricke, Johannes Rübenthaler, Wolfgang G. Kunz

**Affiliations:** ^1^ Department of Radiology, University Hospital, LMU Munich, Munich, Germany; ^2^ Department of Radiation Oncology, University Hospital, LMU Munich, Munich, Germany; ^3^ Department of Medicine III, University Hospital, LMU Munich, Munich, Germany; ^4^ Department of Radiology and Nuclear Medicine, University Medical Centre Mannheim, Medical Faculty Mannheim-University of Heidelberg, Mannheim, Germany

**Keywords:** OMD, cost-effectiveness (economics), radiation therapy (radiotherapy), cancer, SABR

## Abstract

**Background:**

In certain malignancies, patients with oligometastatic disease benefit from radical ablative or surgical treatment. The SABR-COMET trial demonstrated a survival benefit for oligometastatic patients randomized to local stereotactic ablative radiation (SABR) compared to patients receiving standard care (SC) alone. Our aim was to determine the cost-effectiveness of SABR.

**Materials and Methods:**

A decision model based on partitioned survival simulations estimated costs and quality-adjusted life years (QALY) associated with both strategies in a United States setting from a health care perspective. Analyses were performed over the trial duration of six years as well as a long-term horizon of 16 years. Model input parameters were based on the SABR-COMET trial data as well as best available and most recent data provided in the published literature. An annual discount of 3% for costs was implemented in the analysis. All costs were adjusted to 2019 US Dollars according to the United States Consumer Price Index. SABR costs were reported with an average of $11,700 per treatment. Deterministic and probabilistic sensitivity analyses were performed. Incremental costs, effectiveness, and cost-effectiveness ratios (ICER) were calculated. The willingness-to-pay (WTP) threshold was set to $100,000/QALY.

**Results:**

Based on increased overall and progression-free survival, the SABR group showed 0.78 incremental QALYs over the trial duration and 1.34 incremental QALYs over the long-term analysis. Treatment with SABR led to a marginal increase in costs compared to SC alone (SABR: $304,656; SC: $303,523 for 6 years; ICER $1,446/QALY and SABR: $402,888; SC: $350,708 for long-term analysis; ICER $38,874/QALY). Therapy with SABR remained cost-effective until treatment costs of $88,969 over the trial duration (i.e. 7.6 times the average cost). Sensitivity analysis identified a strong model impact for ongoing annual costs of oligo- and polymetastatic disease states.

**Conclusion:**

Our analysis suggests that local treatment with SABR adds QALYs for patients with certain oligometastatic cancers and represents an intermediate- and long-term cost-effective treatment strategy.

## Introduction

Metastatic cancers are considered incurable in a variety of tumor entities. The treatment of choice is systemic therapy. The state of oligometastatic disease (OMD) was introduced in the mid 90s as a subcategory of metastatic cancer. With only a limited number of metastases confined to a few organs, this state may represent a less aggressive tumor biology and open the possibility of treatment in a curative intent (1). However, the oligometastatic state is not fully defined and established (2), and studies regarding treatment are still unfolding (3).

Treatment options include ablative surgery, stereotactic ablative radiotherapy (SABR) and other local ablative procedures like thermal ablation and radioablation, which show different efficacy depending on anatomic location (4). Considering treatment of several metastases in different locations with particularities of their anatomy and composition, SABR has proven to be a targeted treatment option with only few side effects (5, 6) and sufficient local tumor control (7).

The SABR-COMET trial is one of the first phase II trials to compare treatment of patients with one to five metastases of varying tumor entities with standard care (SC) to additional SABR (SABR) (8). The trial demonstrated that combined treatment extended progression-free survival (PFS) and overall survival (OS), all while maintaining quality of life (QoL).

Given this new local treatment option, our aim was to determine the cost-effectiveness of SABR compared to SC, taking into account PFS, OS and QoL.

## Methods

### Model Structure

Our analysis followed recommendations of the Second Panel on Cost-Effectiveness in Health and Medicine (9). We developed a partitioned survival model using decision-analytic software (Treeage Healthcare Pro 2020, Version 20.1.2-v20200326; Treeage, Williamstown, MA) to assess the cost-effectiveness of SABR versus SC over the trial duration of 6 years, using a cycle length of 1 month. Furthermore, long-term survival data was obtained from the Surveillance, Epidemiology, and End Results (SEER) Program (10). The partitioned survival analysis model allows to simulate a patient cohort over time as patients advance along mutually exclusive health states. During each cycle, patients could therefore remain in the oligometastatic state, progress to the polymetastatic disease (PMD) state or die. The only absorbing state was death.

### Model Input Parameters

#### Progression and Survival Probabilities

**Figure 1:**
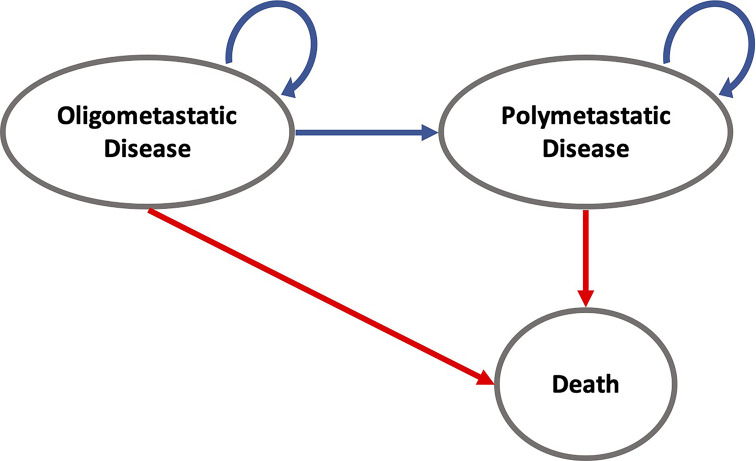
State-transition diagram for modeling cost and effectiveness for the SABR and SC strategies over time intervals. For example, patients in the oligometastatic disease state can either remain in the oligometastatic state, transition to the polymetastatic disease state, or die. Death is an absorbing state and will discontinue the individual simulation.

All individuals started in the oligometastatic state. Monthly overall and progression-free survival rates were derived from the Kaplan-Meier analysis of the SABR-COMET trial (**Supplementary Figure 1**). Therefore, no adjustment for the age-related death rate was necessary. For modeling long-term survival, we referred to the Surveillance, Epidemiology, and End Results Program (SEER) using the SEER*Explorer. OS data were pooled from the database for the metastatic stage of the most frequent cancer entities in the SABR-COMET trial (breast, colorectal, lung, prostate) and fitted with respect to the proportion in the study population. The OS course in the SEER data was used to extrapolate the trial OS curve beyond the trial period. In detail, the curve was expanded beginning from the latest reported OS percentage from the trial and continued with the SEER survival curve at that same percentage. Because of missing data in terms of PFS, we also applied this data to extrapolate the long-term course of PFS; for this we additionally assumed the same proportionality of OS to PFS as in the SABR COMET trial (**Supplementary Table 1**). An overview of the model structure is shown in **Figure 1**.

#### Costs

The analysis was performed in a United States setting from a health care perspective. The ongoing treatment costs for standard care of OMD and PMD states were derived from Reyes et al. (11) and accumulated. These accumulated cost data were used to reflect average annual health expenditures for the patient population that was investigated in the SABR-COMET trial. It further allowed to model the differences in therapy costs during the time intervals spent in the OMD or PMD state. 55% of patients in the SABR group as well as 63.6% of patients in the SC group received systemic therapy. Because of missing information in terms of drug administration, costs were distributed proportionally in the OMD and PMD group. Costs for single treatment of SABR were pooled from assorted papers comprising different fraction numbers and localization of treatment (12–16). Costs for palliative radiotherapy were derived from Medicare coverage data (17).

23 patients in the SC group and 16 patients in the SABR group obtained salvage radiotherapy. 9 patients of the SABR group received additional salvage SABR. Total costs for additional radiotherapy were accumulated per group and factored in as cost items at the beginning of the simulation as data concerning the time of administration was not available; this approximation will slightly alter the costs as these would not be discounted before the actual time of administration. An additional cumulative single time cost was added for the last 180 days of treatment before death (18).

Therapy-related adverse events higher than or equal to grade 2 occurred in 19 patients in the SABR group and 3 patients in the SC group. Costs for treatment (19–21) and disutility (22–26) were pooled from the literature and added as one-time cost and disutility at the beginning of the analysis. An overview of the input parameters is given in **Table 1**. An annual discount of 3% for costs was implemented in the analysis according to current recommendations (9). All costs were adjusted to 2019 US Dollars according to the United States Consumer Price Index.

**Table 1:** Detailed Model Input Parameters.

Model Input	Base Case Value	Range for Sensitivity Analysis*	Distribution	Reference
**Initial Probabilities**				
oligometastatic state	1			Palma et al. (8)
polymetastatic state	0			
death	0			
**Survival Probabilities**				
OS for SC				Palma et al. (8)
1st year	0.88			
2nd year	0.58			
3rd year	0.38	± 15%	ß	
4th year	0.18			
5th year	0.18			
6th year	0.18			
OS for SABR				
1st year	0.88			
2nd year	0.69			
3rd year	0.62			
4th year	0.52			
5th year	0.42			
6th year	0.42			
PFS for SC				
1st year	0.19			
2nd year	0.13			
3rd year	0.07			
4th year	0.04			
6th year	0			
5th year	0			
PFS for SABR				
1st year	0.5			
2nd year	0.38			
3rd year	0.3			
4th year	0.21			
5th year	0.18			
6th year	0.18			
**Health Care Costs**				
**Annual costs for metastatic disease**				
cumulative	$ 97,440	$ 77,952 - 116,928	y	adapted from Reyes et al. (11)
**Annual costs for progressive metastatic disease**				
cumulative	$ 189,840	$ 151,872 - 227,808	y	adapted from Reyes et al. (11)
**End of life costs**				
Last 180 Days	$ 19,174	$ 15,339 - 23,009	y	Bekelman et al. (16)
**Palliative RT costs**				
unit costs	$ 11,070	$ 8,856 - 13,284	y	Agarwal et al. (17)
**SABR costs**				
cumulative	$ 11,700	$ 8,190 - 14,040	y	Hess et al. (12); Kim et al., 2015; Lanni et al. (14); Shah et al. (15); Kim et al., 2016
**Utilities**				
OMD	0.82	0.70 - 0.90	ß	Palma et al. (8) calculated from Teckle et al. (27)
PMD	0.59	0.50 - 0.70	ß	Lloyd et al. (28); Lee at al. (29); Farkilla et al. (30); Petrou and Campbell (31); Llyod et al. (29), Hudgens et al. (32); Paracha et al. (33); Paracha et al. (26); Nafees et al. (24)
**Adverse Events**				
Disutility	SABR: -0.002 SC: -0.0008	± 10%	ß	Palma et al. (8) Hagiwara et al. (22); Chouaid et al. (23); Wehler et al., 2018; Paracha et al. (26)
Treatment costs	SABR: $ 1,443 SC: $ 997	SABR: $ 1,154 - 1,732 SC: $ 798 - 1,196	y	Palma et al. (8) Wong et al. (19) Copley-Merriman et al. (20); Ting et al. (21)

Detailed model input parameters. Survival probabilities and utility for OMD were derived from the SABR-COMET trial. All costs, transitions probabilities for long term survival as well as utility for PMD and disutility from adverse events were derived from the literature. Ranges for deterministic sensitivity analysis were determined by the 95% confidence interval of the initial probabilities and by ±20% for costs. For PSA y-distribution for costs and ß-distribution for utilities was applied. All costs were converted to 2019 USD. *The minimum and maximum values for ranges were derived from reported 95% confidence intervals or from calculated 95% confidence intervals with the use of variance estimates as available.

#### Utilities

Therapy effectiveness was measured in quality-adjusted life years (QALYs), calculated by multiplying years spent in OMD and PMD states by assigned utility weights. Utility weights for OMD were obtained from the FACT-G-Score used in the SABR-COMET trial and converted to EQ-5D according to Teckle et al. (27). Utility weights for PMD were derived from the literature (24, 26, 28–34). A discount of 3% for utilities was implemented in the analysis (9).

### Cost-Effectiveness Analysis

Treatment strategies were compared in terms of net monetary benefits, incremental costs, incremental effectiveness, and incremental cost-effectiveness ratios (ICERs). The willingness-to-pay was set to $100,000 per QALY as in recent studies (35). Net monetary benefits combine costs and effectiveness in one measure: net monetary benefit = (effectiveness × willingness-to-pay) minus costs.

### Sensitivity Analysis

We used comprehensive deterministic and probabilistic sensitivity analysis (PSA) to test the robustness of the model. Deterministic one-way sensitivity analysis was performed to identify variables that significantly influence the model outcomes. The ranges for deterministic sensitivity analysis were determined by the 95% confidence interval of the initial probabilities and by ±20% for costs. Moreover, PSA allows simultaneous alteration of multiple model input parameters using distributions according to probability density functions for second order Monte Carlo simulation runs (n=10,000) (36). The model input parameters were assigned appropriate distributions as indicated in **Table 1**. Utilities were varied with a beta distribution. Treatment costs were modeled by gamma distribution. Beta distributions were used for disutilities as well as PFS and OS data.

## Results

### Base Case Analysis

In the base case analysis of the total study population over the trial duration of 6 years, SABR led to an increased effectiveness of 0.78 QALY at increased costs of $1,133. The ICER was $1,446 per QALY. When additional long-term SEER data were applied, SABR led to an increased effectiveness of 1.34 QALY at additional costs of $52,180. The corresponding ICER was $38,874 per QALY. Adverse events only had a minor effect on our results with a loss of 0.002 QALYs for SABR and 0.0008 QALYs for SC. Incremental costs for treatment of adverse events amounted to $1,443 for the SABR group and $997 for the SC arm.

### Deterministic Sensitivity Analysis

**Figure 2:**
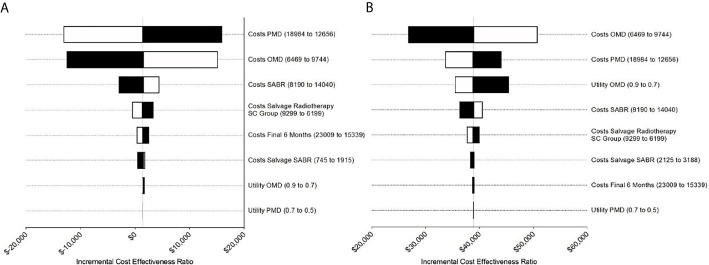
Tornado diagrams for the sensitivity analysis during **(A)** the trial duration and **(B)** long-term simulation extrapolated based on SEER survival data. **(A)** Costs for PMD and OMD demonstrated the strongest impact on ICER during trial duration. **(B)** For long-term analysis costs for PMD influenced ICER the most followed by costs for PMD and utility for OMD.

**Figure 3:**
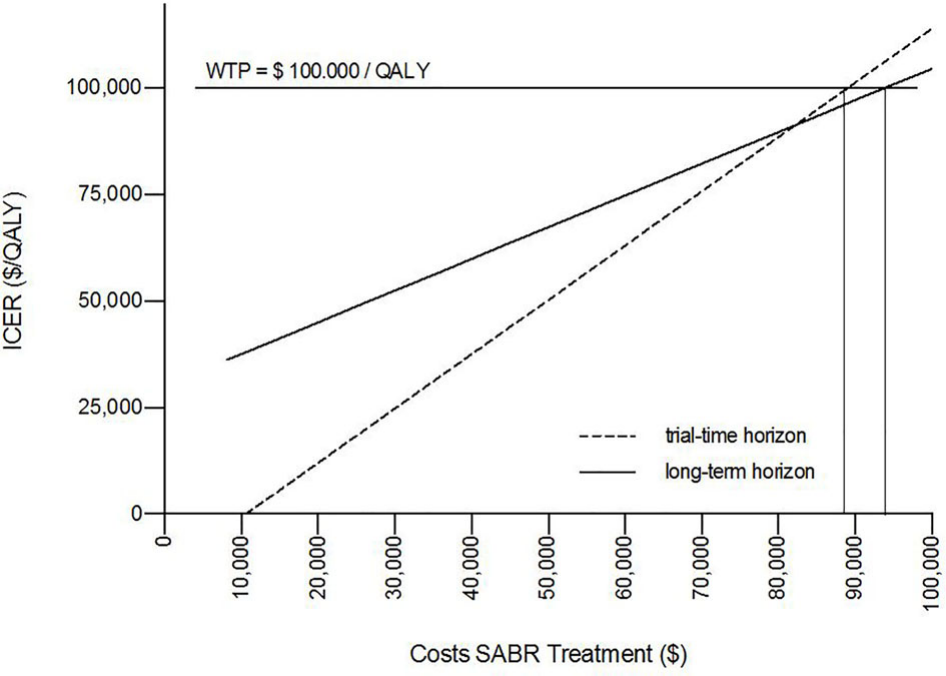
One-way sensitivity analysis proved cost-effectiveness for SABR up to unit costs of $88,696 over the trial duration and $93,750 for long-term survival for a willingness-to-pay (WTP) threshold of $100,000/QALY.

The results of the deterministic one-way sensitivity analysis are presented in **Figure 2**. Costs of systemic therapy of PMD and OMD possessed the strongest impact on ICER regarding the trial duration as well as costs of OMD state on long-term survival. Higher costs of OMD state and lower costs of PMD led to unfavorable ICER values whereas lower costs for therapy of OMD state and higher costs of PMD state led to favorable ICER values. These effects were reversed for the SC strategy. SABR remained cost-effective even when the costs for SABR and salvage SABR were increased 7.6 times during the trial duration and stayed cost-effective when raised up to 8 times for long-term survival (see **Figure 3**).

### Probabilistic Sensitivity Analysis

Overall, SABR was cost-effective in 100% of Monte Carlo simulation runs with an ICER of $1,105 per QALY during the trial duration and $38,740 per QALY for long-term survival in 99.95% of Monte Carlo simulation runs, indicating robustness of the model. In 47% of simulations, SABR was the dominant strategy when analyzed with SABR-COMET data, meaning that it provided better outcomes at lower costs.

The mean incremental effectiveness was positive, meaning that SABR on average led to increased QALYs. Moreover, the mean values for the ICERs were below the willingness-to-pay threshold. The detailed results of the PSA are shown in **Table 2**.

**Table 2:** Cost-effectiveness analysis results.

Trial duration							
Patient group	Cost ($)	IC ($)	Effect (QALY)	IE (QALY)	NMB ($)	ICER	Acceptability
						($/QALY)	at WTP (%)
							
SABR	304,459	1,105	2.58	0.78	-46,000	1,412	100
SC	303,354		1.80		-123,149		
**Long-term analysis**							
**Patient group**	**Cost ($)**	**IC ($)**	**Effect (QALY)**	**IE (QALY)**	**NMB ($)**	**ICER**	**Acceptability**
						**($/QALY)**	**at WTP (%)**
SABR	403,149	52,072	3.37	1.34	-66,632	38,740	99.95
SC	351,077		2.02		-148,975		

Results of cost-effectiveness analysis. SABR proved to be cost effective over the trial duration as well as long-term analysis with an ICER of $1,405 and $38,740 respectively. The willingness-to-pay was set to $100,000 per QALY. SABR, stereotactic ablative radiotherapy; SC, standard care; NMB, net monetary benefit; ICER, incremental cost-effectiveness ratio; IC, incremental cost; IE, incremental effectiveness; WTP, willingness-to-pay threshold.

## Discussion

This study evaluated the economic impact of SABR in the treatment of oligometastatic cancer patients. The analysis indicates that SABR is a cost-effective treatment option compared to SC alone. Additional costs of SABR were partly amortized due to longer progression-free survival in the OMD state, which was accompanied with lower treatment costs of systemic treatment. As expected, DSA demonstrated a relevant impact of treatment costs on the ICER. Yet even with an increase in SABR treatment costs up to about sevenfold, the SABR treatment strategy remained cost-effective.

The SABR-COMET trial is the first basket study to prove survival benefits of SABR treatment in patients with OMD across different cancer entities. Previous cost-effectiveness analysis indicated cost-effectiveness for SABR in oligometastatic prostate cancer and NSCLC (37, 38). Recently, two economic analyse have also analyzed the cost-effectiveness of the SABR-COMET trial (39, 40). Kumar et al. (39) assessed that treatment with SABR is cost-effective in 99.8% of cases at a WTP threshold of $100,000 per QALY, with an ICER of $28,906 per QALY in a US health care setting after a 10-year horizon. Qu et al. (40) showed that SABR is cost-effective over a lifetime horizon in 97% of cases at a WTP threshold of $100,000 per QALY with an ICER of $54,564 per QALY. Kumar et al. used SEER data for long-term analysis over 10 years in total with an increased ICER of $79,406 per QALY if costs for treatment were continued. A detailed comparison of methods and results of these studies is provided in **Table 3**. These data provide external validation and demonstrate robustness of the cost-effectiveness of SABR. Similar to our analysis, Kumar et al. showed cost-effectiveness for SABR up to a 10-fold increase in treatment costs.

**Table 3:** Comparison of SABR-COMET cost-effectiveness analysis.

	Mehrens et al.	Kumar et al.	Qu et al.
Region	US	US	Canada/US
Year	2019	2019	2018
Perspective	healthcare	healthcare/societal	healthcare
Model	PSA	Markov	Markov
Duration	16 years	10 years	20 years
Cycle length	monthly	monthly	3 months
WTP	100,000 USD	100,000 USD	100,000 CAD
Discount	3%	3%	1.50%
Analysis	BCS/DSA/PSA	BCS/DSA/PSA	BCS/DSA/PSA
**Input**			
Survival data	SEER	SEER	Weibull
Cost SABR	11,700 USD/treatment	12,242 USD/treatment	8,378 CAD/ metastasis (1-5)
Cost SC (annually)	97,440 USD 189,840 USD (cancer progression)	96,468 USD 185,436 USD (cancer progression)	chemotherapy 20,813 CAD base cost 14,510 CAD base cost terminal 94,760 CAD
**Results (healthcare)**			
Total cost			
SABR	403,149 USD	460,161 USD	169,693 CAD
SC	351,007 USD	405,901 USD	135,452 CAD
Effectiveness			
SABR	3.37	4.84	2.77
SC	2.02	2.96	1.85
ICER	38,740 USD	28,906 USD	37,157 CAD 54,564 USD
Acceptability SABR	99.95%	99.8%	97%
Miscellaneous	SABR cost-effective until 93,750 USD	SABR cost-effective until 145,688 USD cost-effective for a hazard ratio from 0.3 until 0.76	to remain cost-effective, the HR must decrease by approx. 0.047 for each additional metastasis

Comparison of different cost-effectiveness analysis of the SABR-COMET trial. Results stated are from a healthcare perspective and only long-term survival data were compared. Currency as well as year of the respective analysis were not adapted. Our study demonstrated similar results as the analysis of Kumar et al. Input parameters as well as results from Qu et al. differed from our study as well as from Kumar et al. In probability sensitivity analysis SABR was cost-effective in nearly all of the iterations. PSA, Partitioned survival analysis; BCS, Base case scenario; DSA, Deterministic sensitivity analysis; USD, US-Dollar; CAD, Canadian Dollar.

In contrast to our study, Kumar et al. assumed treatment with SC for all patients and did not include costs for salvage or palliative radiotherapy. Qu et al. used data directly from the SABR-COMET trial, which is not publicly available in its entirety. Moreover, the discount rate was adapted according to Canadian guidelines for the Economic Evaluation of Health Technologies with 1.5% and not 3% as in Kumar et al. and our study. Qu et al. report a non-linear relationship between the number of lesions and the PFS hazard ratio (HR) with the need of decreasing the HR by 0.047 for each additional metastasis to maintain cost-effectiveness for SABR.

Further studies including phase III trials are required to validate the results. Several studies are ongoing at the moment. These include the phase III of the SABR-COMET trial, namely SABR-COMET-3 (41) and SABR-COMET-10 (42), investigating the impact of SABR on patients with 1-3 metastases or 4-10 metastases respectively. By analyzing these two subpopulations, Palma et al. will help to clarify the uncertainty up to which number of metastases patients benefit from SABR. Further phase III trials include the SARON study comparing SC versus SABR and SC for oligometastatic NSCLC with 1-5 metastases in up to a maximum of 3 organs (43), NRG-BR002 investigating systemic therapy versus SABR or surgery combined with systemic therapy in breast cancer with less than 4 metastases (44), and the HALT trial examining the effect of SABR under tyrosine kinase inhibitor (TKI) therapy versus TKI treatment alone in metastatic disease with equal to or less than 3 sites of metastases (45).

The study results should be interpreted with knowledge of the following limitations. First, the current state of evidence on SABR in OMD is still limited by the sample size of the underlying trial; current phase III trials are ongoing. Second, the FACT-G score was stated only for whole populations of study groups. No distinction was made between progression-free and progressive patients. Data on progression-related decrease in QoL were not publicly available from the SABR-COMET study. Third, no information was provided concerning which patients received systemic therapy. Therefore, in our analysis we used the same percentage for treatment with systemic therapy in the progression-free as well as the progressive patient group to avoid introducing any bias. Fourth, because of rapidly changing treatment regimens, specifying a cost for systemic treatment may remain a source of inaccuracy.

Fifth, missing information on which treatment was administered and the inclusion of diverse tumor entities represents a challenge for precise estimation of costs for systemic cancer treatment. This may influence cost-effectiveness as one-dimensional sensitivity analysis demonstrated a great impact of costs for systemic treatment on the ICER. We therefore chose a restrictive approach for our cost-effectiveness analysis, which still indicated cost-effectiveness for the SABR group. Sixth, long-term survival data was obtained from SEER-Program with only OS being available. We deployed these data to also model PFS. Moreover, changes in systemic therapy with more efficient treatments (46, 47) as well as technical advances in planning and performing SABR with accompanying reduction of costs (7) have to be taken into account to obtain an authentic cost estimate in the future.

In conclusion, local treatment with SABR adds QALYs for patients with oligometastatic disease across selected cancer entities in SABR-COMET and represents an intermediate- and long-term cost-effective treatment strategy.

## Data Availability Statement

Publicly available datasets were analyzed in this study. This data can be found here: https://pubmed.ncbi.nlm.nih.gov/32484754/ DOI: 10.1200/JCO.20.00818.

## Author Contributions

DM: Investigation, Data Curation, Formal analysis, Writing - Original Draft, Software. MU: Validation, Writing - Review and Editing. SC: Validation, Writing - Review and Editing. K-MN Validation, Writing - Review and Editing. FM: Validation, Writing - Review and Editing. CW: Validation, Writing - Review and Editing. MF: Validation, Writing - Review and Editing. MW: Validation, Writing - Review and Editing. MS: Validation, Writing - Review and Editing. JeR: Validation, Writing - Review and Editing. JoR: Validation, Writing - Review and Editing. WK: Conceptualization, Methodology, Validation, Supervision, Project administration. All authors contributed to the article and approved the submitted version.

## Conflict of Interest

The authors declare that the research was conducted in the absence of any commercial or financial relationships that could be construed as a potential conflict of interest.
