# Supplementary material for: Cost-Effectiveness Analysis of Local Treatment in Oligometastatic Disease
Source: Front Oncol. 2021 Jun 15;11:667993. doi: 10.3389/fonc.2021.667993 (PMC8239286; doi:10.3389/fonc.2021.667993)

Supplement

Table 1. Long-Term Survival Data

Overall survival			Distribution
	SABR	SC	ß
7th year	0.26	0.1	
8th year	0.19	0.07	
9th year	0.16	0.05	
10th year	0.13	0.04	
11th year	0.12	0.03	
12th year	0.11	0.03	
13th year	0.10	0.02	
14th year	0.09	0.02	
15th year	0.08	0.02	
16th year	0.08	0.02	
**Progression-free survival**			**Distribution**
	SABR	SC	ß
7th year	0.11	0	
8th year	0.08	0	
9th year	0.07	0	
10th year	0.06	0	
11th year	0.05	0	
12th year	0.05	0	
13th year	0.04	0	
14th year	0.04	0	
15th year	0.04	0	
16th year	0.03	0	

For longtime survival we referred to the Surveillance, Epidemiology, and End Results Program (SEER) using the SEER*Explorer. OS data were pooled from the database for metastatic stage of the most frequent cancer entities in the SABR-COMET trial (breast, colorectal, lung, prostate) and fitted in respect to the proportion in the study population.

Figure 1. Comparison of model progression-free and overall survival output (left) to Kaplan-Meier curves from the SABR-COMET trial (right)

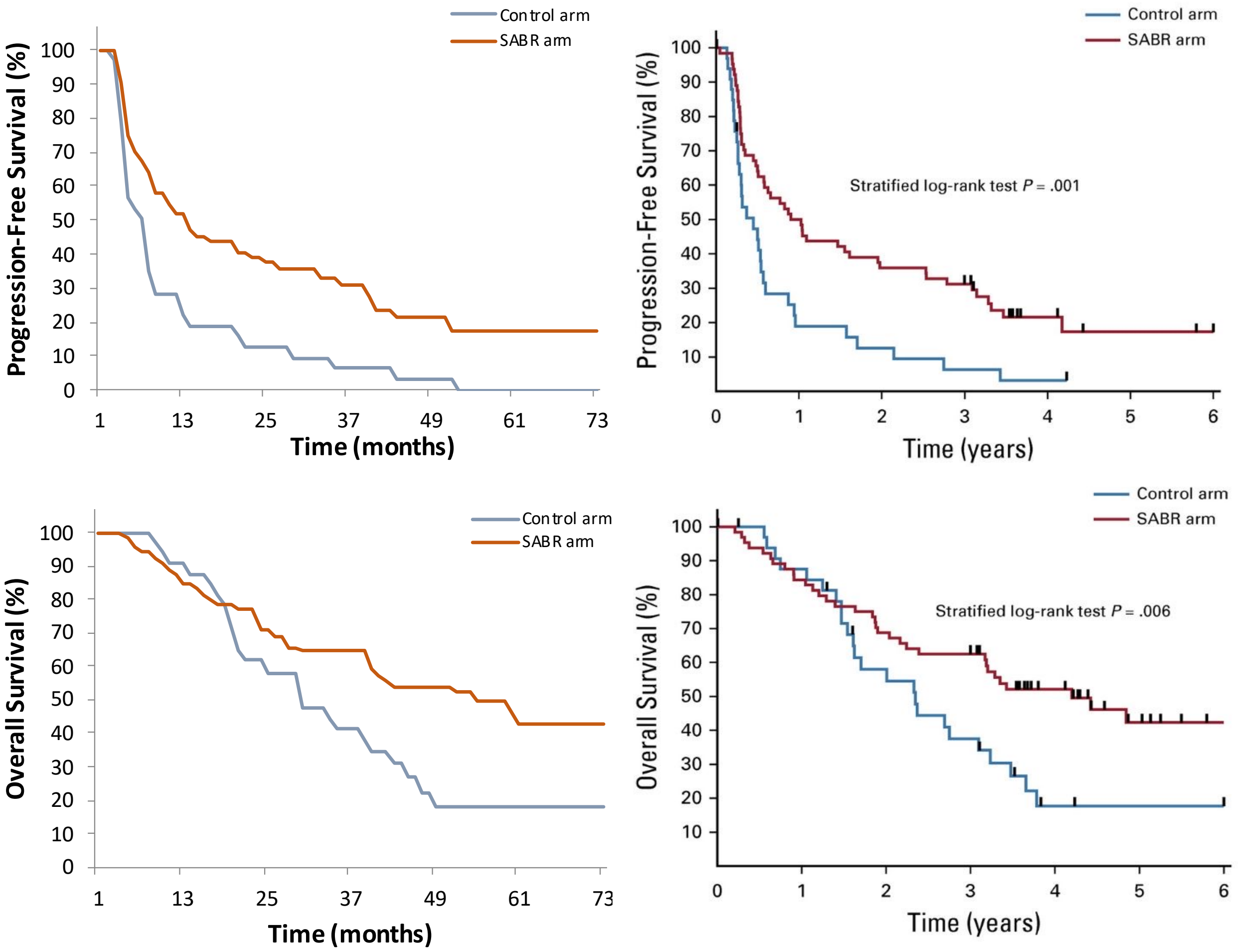

Supplement: Supplementary file 1 [file DataSheet_1.pdf]
